# Natural Pig Plasma Immunoglobulins Have Anti-Bacterial Effects: Potential for Use as Feed Supplement for Treatment of Intestinal Infections in Pigs

**DOI:** 10.1371/journal.pone.0147373

**Published:** 2016-01-29

**Authors:** Chris J. Hedegaard, Mikael L. Strube, Marie B. Hansen, Bodil K. Lindved, Allan Lihme, Mette Boye, Peter M. H. Heegaard

**Affiliations:** 1 National Veterinary Institute, Technical University of Denmark, Frederiksberg, Denmark; 2 Upfront Chromatography A/S, Copenhagen, Denmark; Indian Institute of Science, INDIA

## Abstract

There is an increasing demand for non-antibiotics solutions to control infectious disease in intensive pig production. Here, one such alternative, namely pig antibodies purified from slaughterhouse blood was investigated in order to elucidate its potential usability to control post-weaning diarrhoea (PWD), which is one of the top indications for antibiotics usage in the pig production. A very cost-efficient and rapid one-step expanded bed adsorption (EBA) chromatography procedure was used to purify pig immunoglobulin G from slaughterhouse pig plasma (more than 100 litres), resulting in >85% pure pig IgG (ppIgG). The ppIgG thus comprised natural pig immunoglobulins and was subsequently shown to contain activity towards four pig-relevant bacterial strains (three different types of *Escherichia coli* and one type of *Salmonella enterica*) but not towards a fish pathogen (*Yersinia ruckeri*), and was demonstrated to inhibit the binding of the four pig relevant bacteria to a pig intestinal cell line (IPEC-J2). Finally it was demonstrated in an *in vivo* weaning piglet model for intestinal colonization with an *E*. *coli* F4+ challenge strain that ppIgG given in the feed significantly reduced shedding of the challenge strain, reduced the proportion of the bacterial family *Enterobacteriaceae*, increased the proportion of families *Enterococcoceae* and *Streptococcaceae* and generally increased ileal microbiota diversity. Conclusively, our data support the idea that natural IgG directly purified from pig plasma and given as a feed supplement can be used in modern swine production as an efficient and cost-effective means for reducing both occurrence of PWD and antibiotics usage and with a potential for the prevention and treatment of other intestinal infectious diseases even if the causative agent might not be known.

## Introduction

Antibodies are major effector molecules of the vertebrate adaptive immune system. In mammals, circulating antibody isotypes are predominantly immunoglobulin (Ig) G, and to a lesser extent IgA and IgM [1]. Maternal IgG is provided to the offspring ensuring an adequate level of circulating antibodies in the neonate by different routes depending on the species. Thus, among mammals, primate neonates are born with circulating IgG obtained via the placenta in the foetal stage, whereas ruminants and piglets acquire IgG through colostrum perinatally through an intestinal transport mechanism specifically working in the first 24 hours after birth allowing entry of ingested maternal IgG from the gut to the circulation [2]. In addition, mother’s milk provides all mammals in the suckling stage with oro-gastric protection (lactogenic immunity) against pathogens until the offspring matures and becomes able to produce antibodies both at mucosal surfaces and in the circulation [2–5]. In intensive pig production, piglets are weaned early when still immunologically incompetent (3–4 weeks of age), and they are therefore susceptible to intestinal (mucosal) infections including enterotoxigenic *Escherichia coli* (ETEC) [6] and depend on maternal antibodies for protection against such infections. ETEC infections are major contributing factors to the multifactorial disease post weaning diarrhoea (PWD) demanding excessive antimicrobial use in husbandry [7, 8] resulting in increasing incidence of antimicrobial-resistant bacteria posing a great threat to human health [8–10]. Vaccines may provide protection against specific pathogens, however as diseases such as PWD often has a multifactorial aetiology [11], generating vaccines against such diseases is a complicated matter. Furthermore, efficient mucosal immunity is not easily induced [12]. With the exception of one commercially available, orally delivered *E*. *coli* F4-specific vaccine against PWD used in Canada for a number of years [13] and recently approved by the European Medicines Agency in Europe [14], no vaccines are available for use against PWD.

In contrast, spray-dried porcine plasma (SDPP) is widely used in intensive swine production as a feed additive resulting in increased average daily growth (ADG) and a reduced feed/growth ratio (FGR) of weaners, especially in conventional (as opposed to SPF) herds (reviewed in [15]). In challenge experiments weaners fed SDPP and challenged with relevant pathogens showed a significant increase in ADG as well as a significant reduction in FGR one week after weaning in comparison to a challenged control group not receiving SDPP [15]. The active part of SDPP responsible for this effect has been proposed to be natural IgG antibodies [16], which are present in high concentrations in SDPP and are presumed to be responsible for the observed reduction in incidence of ETEC infections by SDPP in experimental weaner piglet models for PWD [17, 18].

Hence, with the known protective role of maternal antibodies and the evidence pointing to IgG being the active part of SDPP we hypothesise that weaner piglets would benefit from receiving a supplement of purified natural pig antibodies in their diet. These natural antibodies could consequently provide an alternative to antibiotics.

In order to become a viable alternative to antibiotics in the intensive pig production a product based on natural antibodies should be affordable to the users and, therefore the method used to purify the immunoglobulins should be efficient, very economical and able to handle very large volumes of starting material (blood plasma). These are all characteristics of the chromatographic expanded bed adsorption (EBA) procedure which has previously been demonstrated for the efficient purification of immunoglobulins directly from blood plasma [19]. In this study we investigate the potential of natural antibodies purified by EBA chromatography from normal finisher swine blood plasma for binding to pig relevant intestinal pathogens as well as for their inhibition of adhesion to a porcine intestinal cell line *in vitro* and for their inhibition of intestinal colonization in a pig *in vivo* model of *E*. *coli* colonization.

## Materials and Methods

### Purification of pig immunoglobulins

Pig immunoglobulin G was purified from approximately 110 litres concentrate of porcine blood plasma obtained from Daka SARVAL A/S (Lunderskov, Denmark) at UpFront Chromatography A/S (Copenhagen) by high-volume Expanded Bed Absorption (EBA) affinity chromatography with a proprietary absorbent [19]. Before applied in the *in vivo* study (see below), the purified pig IgG (ppIgG) was diluted to 200 mg/ml in PBS.

### Electrophoresis and Western blotting

Samples for SDS PAGE were mixed with 4X LDS Sample buffer and 10X reducing agent (Life Technologies, Taastrup, Denmark). For SDS PAGE Novex Bis-Tris 12%, 1 mm polyacrylamide gels were used in the NuPAGE buffer system according to the manufacturer’s instructions (Life Technologies), loading 20 μl sample in each well. After electrophoresis, gels were stained with Bio-Safe^™^ Coomassie G-250 stain (Bio-Rad, Copenhagen, Denmark).

For immunoblotting, separated proteins were electroblotted from SDS PAGE gels onto nitrocellulose membranes (Millipore, Copenhagen, Denmark) in a Mini Trans-Blot transfer cell including a cooling unit (Bio-Rad) with 12.5 mM Tris, 96 mM Glycine (both from Sigma-Aldrich, Copenhagen, Denmark), 20% Ethanol (VWR—Bie & Berntsen A/S, Herlev, Denmark) pH 8.4 for 1 hour at 150 mA constant current. All subsequent operations were performed at room temperature with shaking. Next, the membranes were blocked in 50 mM Tris, 250 mM NaCl (TBS) with 2% Tween 20 at pH 8.6 for 5 minutes; followed by 3x10 min washes in TBS-T (TBS with 0.1% Tween 20 (Merck, Hellerup, Denmark)). Then, membranes were incubated with polyclonal biotinylated rabbit anti-Pig F(ab)_2_ IgG (LSBio, Copenhagen, Denmark) diluted 1:10.000 in TBS-T for 1 hour followed by 3x10 min washes in TBS-T before the last incubation with alkaline phosphatase-conjugated streptavidin (DAKO, Glostrup, Denmark) 1:3000 in TBS-T (1 hour). After 3x50 ml washes in TBS-T, blots were developed with 4-nitro-blue-tetrazolium/5-bromo-4-chlor o-3-indolyl-phosphate (NBT/BCIP) tablets (Roche, Hvidovre, Denmark) following the manufacturer’s instructions, terminating colour development by washing the blot with several changes of MilliQ water.

### Bacterial isolates

Three *Escherichia coli* strains (O138, O149:F4 and F18) were isolated from faeces of piglets suffering from PWD and *Yersinia ruckeri* was isolated from Enteric Redmouth Disease affected *Oncorhynchus mykiss*. *Salmonella enterica diarizonae* was kindly provided by Gitte Sørensen at DTU Food.

All bacteria were fixed in 0.5% formaldehyde/PBS. Furthermore, *E*. *coli* serotype O138 and *S*. *diarizonae* extracts for ELISA were prepared by treatment with sodium deoxycholate (DOC), as described in [20]; the 0.1% DOC fractions of *E*. *coli* O138 and *S*. *diarizonae* were used in the ELISAs. Bacterial concentrations were determined by CIBA-Corning 254 colorimeter at an optical density of 546 nm.

### Enzyme linked immunosorbent assays (ELISAs)

Generally, for all ELISAs washing steps consisted of four times washes in PBS with 0.05% Tween 20 (PBS-T). PBS-T with 1% bovine serum albumin (Sigma-Aldrich) was used as both blocking and dilution buffer. Coating was done with 100 μl/well at 4°C overnight. Blocking was done with 200 μl blocking buffer shaking for 30 min. at room temperature. Plates were 96 wells flat bottom Maxisorp plates (NUNC, Thermo Scientific, Denmark) and all operations were performed at room temperature unless specified otherwise. Development of plates was done by TMB Plus substrate (Kem-En-Tec, Taastrup, Denmark), 100 μl/well, and stopping colour development by 100 μl 0.5 M H_2_SO_4_ (VWR—Bie & Berntsen A/S). Optical densities of microtiter plates were measured by Thermo Scientific Multiscan EX microplate reader at 450 nm absorbance and the background absorbance at 650 nm was subtracted.

Competitive ELISA for determination of binding of antibodies to specific antigens: wells were coated with DOC antigen extracts in 0.1 M sodium carbonate buffer pH 9.6 at 3.3 μg/ml (*E*. *coli* extract) or 4.8 μg/ml (*Salmonella diarizonae*). After blocking, swine immunoglobulins and horse radish peroxidase (HRP)-conjugated antibodies, either goat polyclonal anti-*E*. *coli* (18-511-245057) or rabbit polyclonal anti-*salmonella* (18-511-245055) (both Genway Biotech. Inc., Hölzel Diagnostika, Cologne, Germany) were diluted in deionised water with 2.5% casein Hammerstein (VWR—Bie & Berntsen A/S) pH 7 (2.5 μg/ml (anti-*salmonella*; 18-511-245055), 5 μg/ml (anti-*E*. *coli*; 18-511-245057)) and incubated for 1 hr. The plates were then washed, developed and read as described above. Each plate held several controls with no swine immunoglobulins added in order to define the uninhibited OD in the remaining sample. From this the % inhibition in a sample was defined as $100-\frac{ODsample}{ODcontrol}*100$.

Whole-cell ELISA for demonstration of antibody binding to bacterial surfaces: wells were coated with fixed bacteria in 0.1 M sodium carbonate buffer pH 9.6 (final OD_546_ = 0.25). After blocking, swine plasma IgG was added in 2-fold dilution series from 10 to 0.02 mg/ml. After 1 hour of incubation and 3 washes in PBS-T, detection antibody (HRP-conjugated rabbit anti- porcine Ig (DAKO, Glostrup, Denmark) diluted 1/7000 or HRP-conjugated goat anti-pig IgG (GGHL-5P; ICL, SMS Gruppen; Rungsted, Denmark) diluted 1/2000) was added and incubated for 1 hour. After washing, plates were developed and measured as described above.

Indirect ELISA for determination of relative avidity of IgG: After coating the wells with *E*. *coli O138* DOC extract in 2-fold serial dilutions starting at 66.6 μg/ml, plates were blocked and subsequently washed, followed by incubation with 6 mg/ml ppIgG (in 2.5% casein pH 7) for 1 hour. Following the wash steps, 100 μl of PBS-T with 0, 2, 4 or 6 M urea was added to designated wells, and after 30 minutes of incubation and shaking wells were washed again, followed by incubation with HRP-conjugated rabbit anti-porcine Ig diluted 1/7000 for 1 hour. The ELISAs were developed and read as described above.

### *In vitro* inhibition of *E*. *coli* adhesion

One day before the assay was carried out, 100 μl (15,000 cells) neonatal porcine jejunum derived IPEC-J2 cells (ACC-701; DSMZ, Braunschweig, Germany) were seeded in each well of a 96-well plate (NUNC) and were allowed to attach for 16 hrs. On the same plate, control wells received 100 μl of the cell culture medium (Advanced DMEM/F12 supplemented with 5% heat-inactivated foetal bovine serum, 2 mM GlutaMAX, 100 units/ml penicillin, 100 μg/ml streptomycin, and 2 μg/ml Fungizone; all purchased at Life Technologies) without IPEC-J2 cells.

*E*. *coli* and *S*. *diarizonae* colonies were retrieved from blood plates and used for inoculating brain-heart broth (agar and media purchased at BD Biosciences, Albertslund, Denmark) cultures incubated overnight with shaking at 37°C. For staining bacteria with Syto24 (Life Technologies), the culture medium was changed to deionised water diluting the cells to OD_546_ 0.16 and Syto24 was added to a final concentration of 6.5 μM. After 30 min., bacteria were centrifuged at 4500 rpm for 5 min. at 4°C (SL40R, Thermo Scientific, Holm & Halby, Brøndby, Denmark) and thereafter washed twice in 4°C deionised water and then twice in 4°C adhesion buffer (12.5 mM HEPES, 141 mM NaCl, 0.5 mM MgCl_2_, 0.15 mM CaCl_2_, 0.1% gelatine (all purchased at Sigma-Aldrich); pH 7.4).

Syto24-stained bacteria were pre-incubated with various amounts of ppIgG (from 100 mg/ml to 0.2 mg/ml, plus controls with no ppIgG) in a total volume of 50 μl for 30 min at 4°C. While the bacteria and immunoglobulins were incubating, the IPEC-J2 cells were placed for 30 min at 4°C; just before addition of pre-incubated bacteria, the IPEC-J2 cell culture medium was substituted with 50 μl 4°C adhesion buffer. Next, 50 μl bacteria in different swine immunoglobulin concentrations were added in parallel series to wells with or without IPEC-J2 cells; the latter wells served as non-adhesion controls. The plates were incubated for 180 min at 4°C. After adhesion, plates were washed three times in 100 μl adhesion buffer, and 100 μl adhesion buffer was added before reading fluorescence at 485/528 nm by means of Synergy HT (BioTek, Holm & Halby, Brøndby, Denmark) using Gen5 software (BioTek).

### *In vivo* study

The offspring of 11 sows were randomly mixed after farrowing, thus avoiding confounding treatment effect with the genetic background. At 3 weeks of age, piglets were additionally allowed access to a custom made feed consisting of 50% cracked wheat and 50% ground oat (NAG, Helsinge, Denmark). The oat was grounded in an Electrolux EFP5100 food processor (Kvickly, Denmark) to ensure even particle size between the main components of the diets. The diets were custom made to avoid enzyme mixtures (such as phytase), as well as antibacterials and heavy metals.

At 28 days of age twenty-four piglets were randomly selected and weaned, and transported to the housing facility and distributed according to their experimental group A, B or C (Table 1). Each pen of 8 piglets was provided with 2.5 kg of oat/wheat-feed each morning during the experiment, and group C was provided with oat/wheat-feed mixed with 160 ml (32 grams) of the IgG product. Water was provided ad libitum from water nibble, and animals were kept in 12/12 hour light/dark conditions with temperature controlled to 15°C and access to a heating lamp.

**Table 1:** Experimental setup of the animal study.

Group	IgG	Infection	Avg. initial weight (kg)	Avg. final weight (kg)
**A**	-	-	8.2	7.7
**B**	-	+	6.8	6.7
**C**	+	+	8.3	8.3

On the day of weaning (day 1), groups B and C were given 15 mL of an oral suspension containing 2x10^10^ colony forming units of *E*. *coli* F4+O149 from an overnight culture grown in Luria-Bertani broth (BD Bioscience). This was repeated on day 2 (the day after weaning).

The animals were inspected daily for signs of diarrhoea and general well-being. Faecal samples were collected on day 1, 3, 5, 7, 9 and 11 post infection. Four randomly selected weaners from each pen were killed at day 11 and the remaining half were killed at day 12 and inspected. Animals were sacrificed by an overdose of pentobarbital and jugular bleeding. The contents of the ileum were taken and stored at -20°C until further analysis.

All animal procedures were approved by the Danish Animal Experiments Inspectorate under the Ministry of Justice (permit number: 2014-15-2934-01048) and animal experiments were conducted in strict accordance with their guidelines. Animals were acquired from a commercial farm near Roskilde, Denmark.

### Quantitative PCR for *E coli* F4 gene

To follow shedding of the *E*. *coli* F4+O149 infection throughout the experiment, a qPCR specific for the F4 gene was performed on faecal samples as described by Ståhl et. al.[21].

### Next generation 16S rDNA sequencing

DNA was purified from the ileum samples and the V1-V2 regions of 16S rRNA gene was amplified by PCR and sequenced as described by Strube et al. [22]. Briefly, the 16S rRNA gene amplicons were submitted to the DTU Multi-Assay Core (https://dmac.cbs.dtu.dk/) and sequenced on the MiSeq platform (Illumina Inc. San Diego, USA). The resulting sequences were then merged, quality filtered and mapped against the RDP-II SSU database [23] using the BION-meta software (Danish Genome Institute, Aarhus, Denmark). The resulting read counts were then analysed on the family level. Data were uploaded to Genbank database: http://www.ncbi.nlm.nih.gov/sra/SRP066524.

### Statistics

qPCR data was fitted for each group with an equation of the form:

E. coli=100*Time* e−OnsetTime*e−Time*e−ClearenceTime, which describes the shedding as a function of time by the onset rate and the clearance rate. These constants were generated by nonlinear regression through the gauss-newton algorithm, and as the estimates of the constants are normally distributed, they could be compared by a t-test.

Sequencing data was analysed by one-way ANOVA followed by Tukeys post-hoc test on log transformed data. Diversity was calculated as the Shannon-index. Multivariate analysis was carried out by non-metric multidimensional scaling (NMDS) with Bray-Curtis distances. The statistical software R v.3.1.0 (Vienna, Austria; http://www.R-project.org/.) was used for the above statistics evaluations.

Two-way ANOVA with Bonferroni post-hoc test was performed for statistical analysis of the ELISA assays by means of GraphPad Prism version 5.00 for Windows, GraphPad Software, San Diego California USA, www.graphpad.com.

## Results

### Characterisation of the Immunoglobulin product

**Fig 1:**
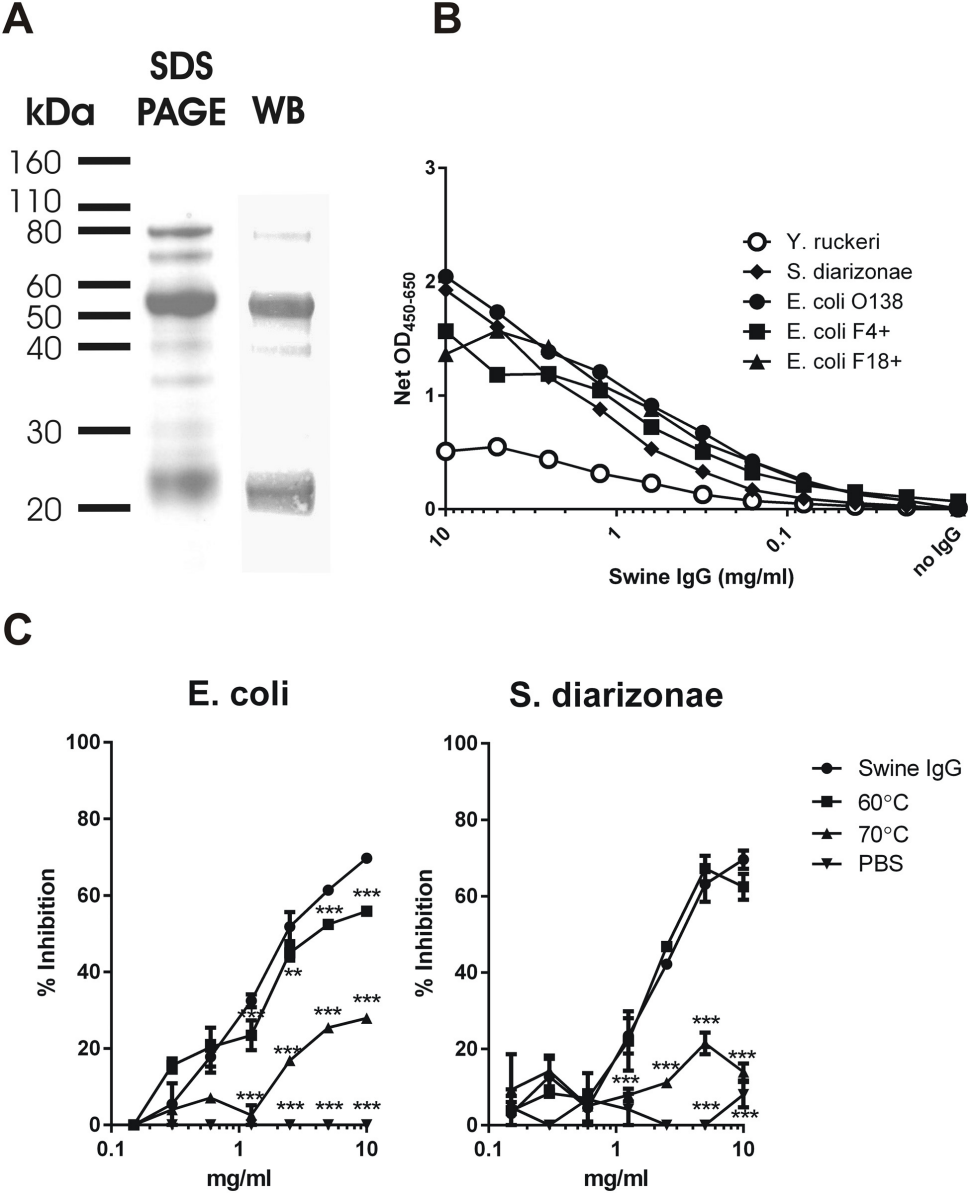
Characterisation of ppIgG. **(A)** Pig plasma IgG product (ppIgG, 1 mg/ml) was run under reducing conditions on 12% SDS PAGE as described in Materials and Methods. In parallel, Western blotting (WB) was performed on the same sample. The blot was developed with biotinylated rabbit anti-pig IgG F(ab)_2_ antibody, followed by alkaline phosphatase-coupled streptavidin (see Materials and Methods). **(B)** Wells were coated with formalin-fixed bacteria (Y. ruckeri, S. diarizonae, *E. coli* O138, O149:F4 and F18+) in separate ELISA plates, and incubated with ppIgG added in serial 2-fold dilutions, and the bound IgG was estimated by using a HRP-conjugated rabbit anti-pig IgG antibody. Data from one typical experiment are presented. **(C)** ppIgG was tested for reactivity against *Escherichia coli* and *Salmonella diarizonae* by competitive ELISA as described (Materials and Methods). ppIgG heated at 60°C or 70°C for 1 hour and PBS were used as control samples. The results are presented as ‘% inhibition’ of the signal obtained in the absence of ppIgG (median values (± median ranges)). Two-way ANOVA (subjected to Bonferonni post-test) was used to observe statistical significance between the treated and untreated ppIgG (***: p<0.01; **: p<0.01).

By SDS PAGE and Western blotting analysis the ppIgG was estimated to consist of approximately 85% pure immunoglobulin (Fig 1A). These impurities are mainly albumin and transferrin.

### Antibody binding to bacteria

An indirect whole-cell ELISA was set up to assess the binding of ppIgG to relevant bacteria. We found that ppIgG bound to the bacterial surface of the three tested *E*. *coli* strains as well as *S*. *diarizonae*, however showed negligible binding to the fish pathogen *Y*. *ruckeri* (Fig 1B). The avidity of ppIgG binding to *E*. *coli* ppIgG was tested by incubating an *E*. *coli* extract with ppIgG at 6 mg/ml and then subjecting it to increasing concentrations of urea for up to 30 minutes. Strong denaturing conditions (6 M urea, 30 minutes) significantly reduced the binding but never more than 35% (S1 Fig). Comparing the binding of IgG from either plasma or the purified IgG in the subsequent eluate to bacterial surface of *E*. *coli* revealed that the purification increased anti-*E*. *coli* titres with a factor 10 (S2 Fig).

A competitive ELISA was used to evaluate the binding activity toward bacteria of the ppIgG as scored by its ability to out-compete a detection antibody having specificity for the bacterium in question, using ELISA plates coated with bacterial extract of either *E*. *coli* or *S*. *diarizonae*. Half-maximal inhibition was obtained with 2.4 mg/ml and 3.4 mg/ml of ppIgG on *E*. *coli* and *S*. *diarizonae* extracts, respectively (Fig 1C). At least 1 mg/ml of ppIgG had to be applied in the competitive ELISA assay to obtain inhibition greater than 20%.

Denaturation of ppIgG by heating to 70°C for 1 hour strongly decreased the binding (to 27% of the binding of native ppIgG; 5 mg/ml ppIgG on *E*. *coli* extract, Fig 1C) highlighting that the binding was dependent on the specific binding activity of ppIgG.

Finally, we investigated the impact of storage on the binding activity of ppIgG. After 65 days of storage, ppIgG showed a significant decrease in binding activity, irrespectively of storage conditions, however when kept at -20°C more than 80% of the native binding activity of ppIgG was retained after 30 days of storage (S1 Table and S3 Fig).

### Inhibition of bacterial adhesion

**Fig 2:**
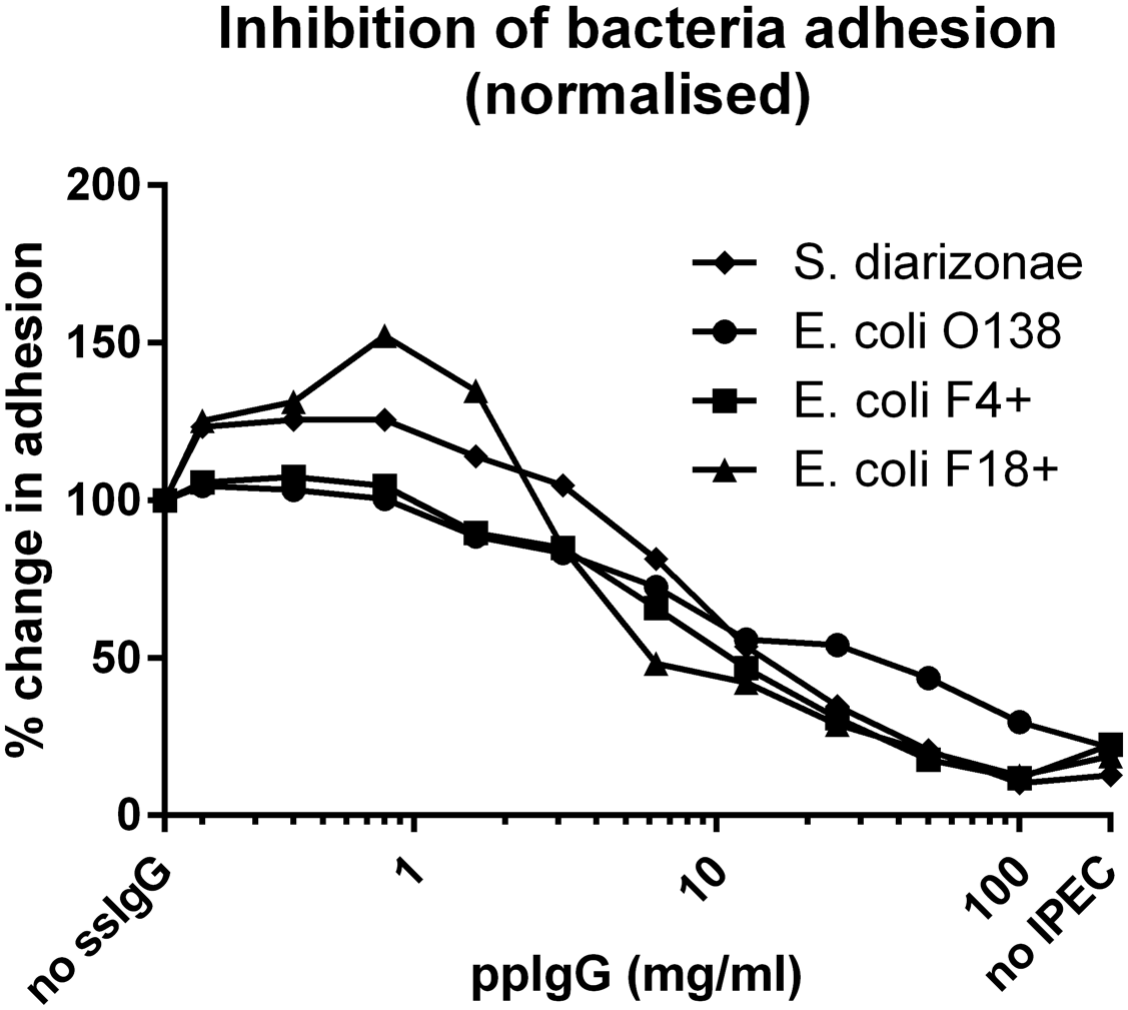
Inhibition of bacterial adhesion to intestinal epithelial cells. Bacteria (*S*. *diarizonae*, *E*. *coli* O138, F4+ or F18+) fluorescently stained as explained in Materials and Methods, and treated with ppIgG in different concentrations (from 100 mg/ml to 0.2 mg/ml) were incubated with IPEC-J2 cells. Binding of bacteria to IPEC-J2 is depicted in relation to non-inhibited bacteria (no ppIgG, set to 100%). One typical experiment is shown.

To investigate if natural pig plasma immunoglobulins could interfere with bacterial adhesion to intestinal epithelium an *in vitro* adhesion-inhibition assay was employed using fluorescently labelled bacteria and a neonatal porcine jejunum derived cell line (IPEC-J2) as a model of the intestinal epithelium. Pre-incubation of fluorescently stained bacteria with ppIgG for 1 hour before adding the bacteria to the IPEC-J2 cells, significantly decreased adhesion of both *E*. *coli* and *S*. *diarizonae* to IPEC-J2 cells. This was observed at 1 mg/ml or more of ppIgG; at 100 mg/ml IgG fluorescence was at background level indicating complete inhibition of adhesion (Fig 2). This indicates that high concentrations of natural pig immunoglobulins interfere with adhesion of both *E coli* and *S*. *diarizonae* to intestinal epithelial cells.

### *In vivo* study

**Fig 3:**
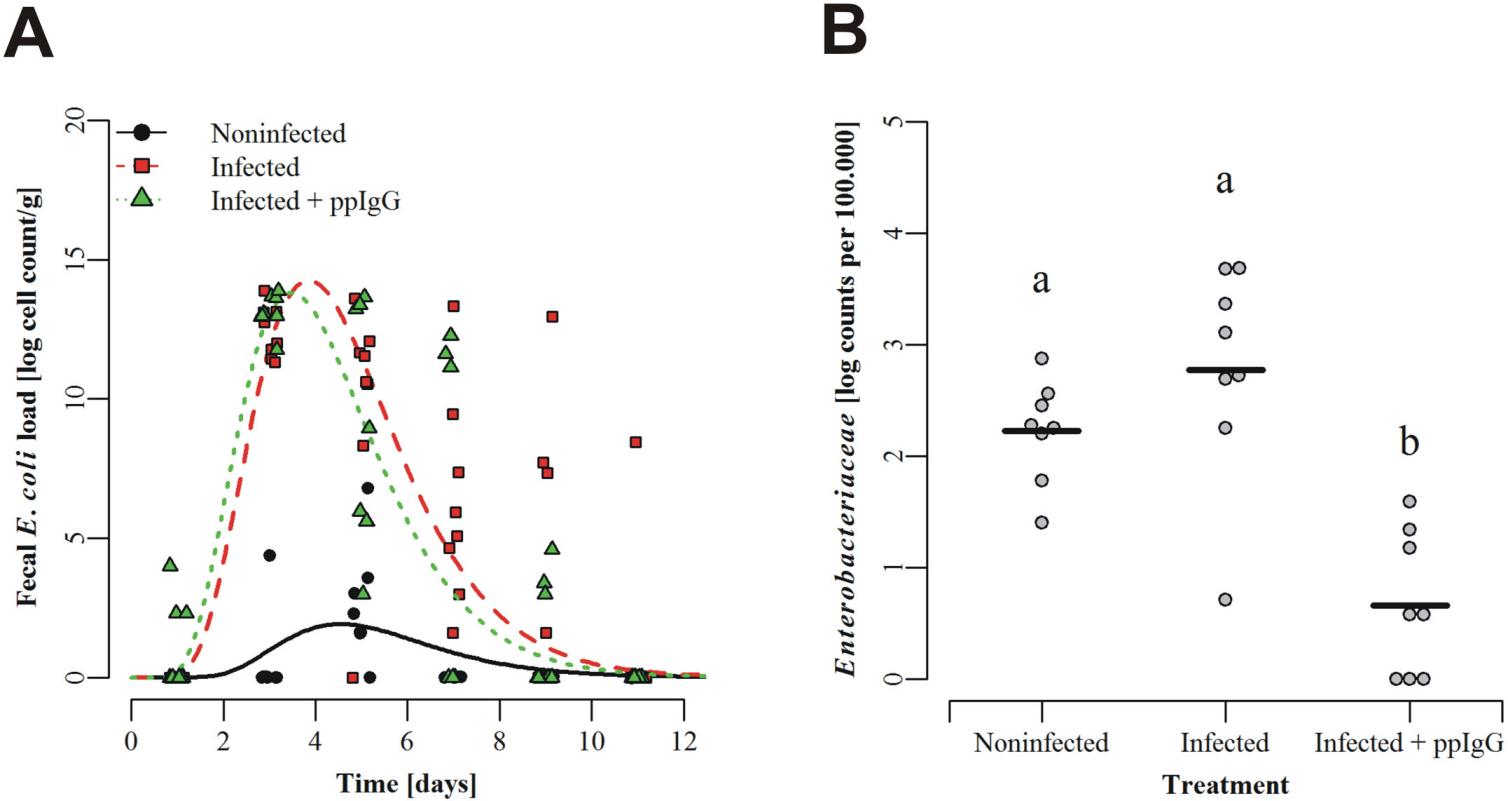
The impact of natural immunoglobulins on enteral *Enterobacteriaceae*. **(A)** Shedding of *E*. *coli* F4+ in an *in vivo* infection model. Animals were infected orally at the day of weaning as well as the day after weaning with *E*. *coli* F4+ (see Materials and Methods), after which faecal shedding was followed with a F4 specific qPCR. Data were log-transformed before analysis by the non-linear equation, see Materials and Methods for further details. **(B)** Ileal content of family *Enterobacteriaceae*. Bacteria were enumerated by NGS sequencing of the 16S rDNA gene in ileal samples obtained at necropsy by the end of the experiment. Values are normalized (per 100,000 reads) and log-transformed read counts. Overall ANOVA p-value < 0.001, different letters denote significantly different values.

**Fig 4:**
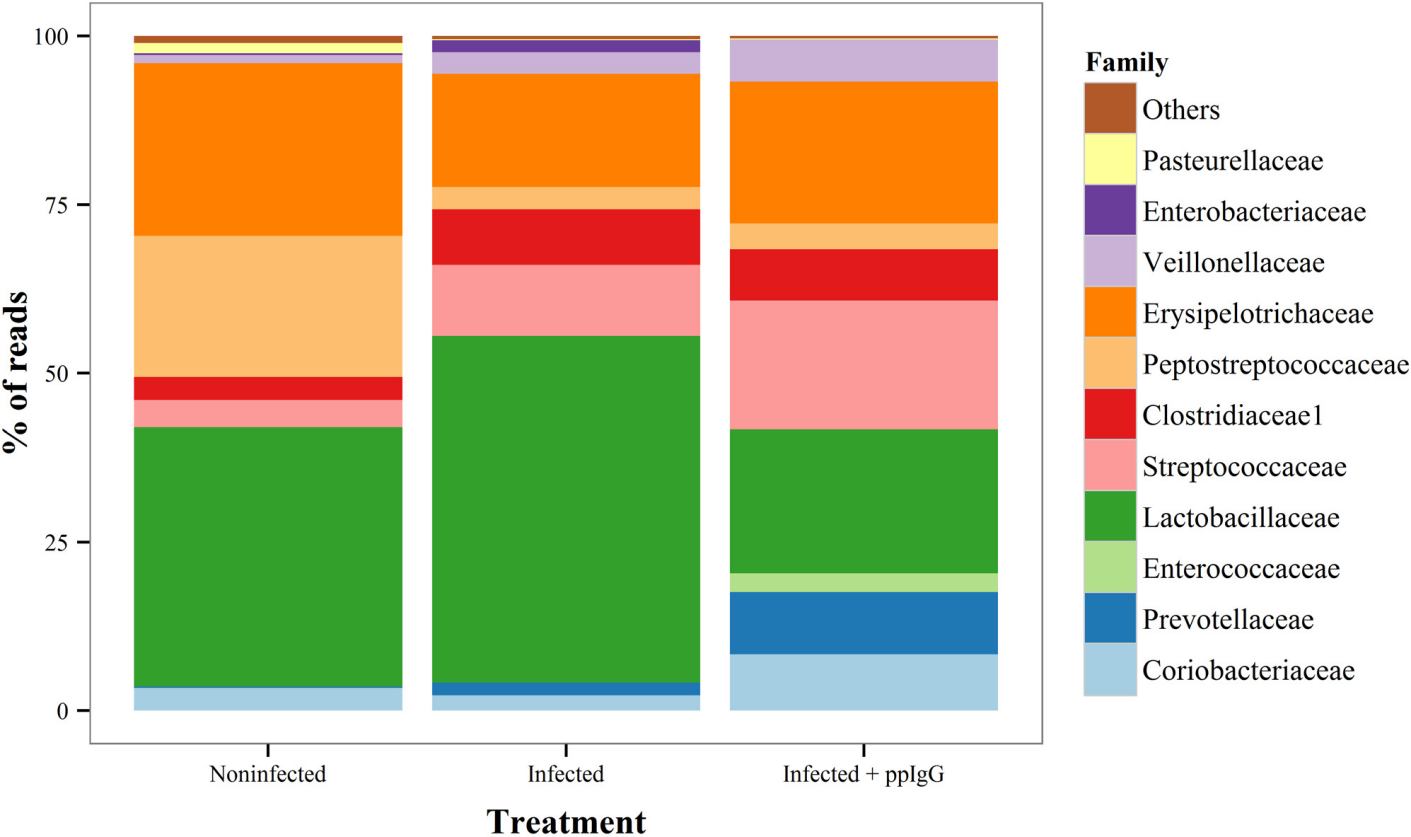
The relative composition of the ileal microbiota on a family level. Bacteria were enumerated by NGS sequencing of the 16S rDNA gene in ileal samples obtained at necropsy by the end of the experiment.

Piglets were infected experimentally with *E*. *coli O149*:*F4* with (group C) and without (group B) ppIgG supplementation in the feed. A non-infected non-ppIgG-feed group (group A) served as a negative control for infection. The feed was generally well tolerated by the piglets; however on average the animals did not gain weight during the course of the study, irrespectively of experimental group (see Table 1) most probably due to a discernible reluctance of the weaners of all groups to feed for the first days after arrival. The infection did not cause clinical disease, however shedding of the *E*. *coli* inoculation strain was 10^5^ and 10^4^ times higher at day 3 and day 5 after infection, respectively, in the two infected groups compared to the control group (Fig 3A), indicating intestinal colonization by the challenge bacterium. The mathematical model describing the shedding pattern shows that the infection was cleared significantly faster in group C (infection + ppIgG) compared to group B (infection only)(p = 0.0007), even if onset of infection was faster in group C than in group B (p = 0.0017). The effect of ppIgG on the intestinal microbial colonisation was further investigated by deep sequencing of the ileal microbiota (Fig 3B). This showed a significantly lowered (p<0.001) colonisation of the family *Enterobacteriaceae* in the ileum as compared to both the non-infected control (group A) and the infected control group (group B, Fig 3B). Collectively the data presented in Fig 3 suggest that ppIgG inhibits adhesion of bacteria from the family *Enterobacteriaceae*, including *E*. *coli*. Conversely, the families *Enterococcoceae* and *Streptococcaceae* were significantly (p<0.01) increased in the ppIgG group compared to both other groups, and compared to the uninfected group only, respectively (Fig 4). *Lactobacillus*, *Bifidobacterium* and other bacterial families generally viewed as health promoting did not differ significantly between groups (Fig 4). Furthermore, the diversity (Shannon Index) was significantly (p<0.05) higher in the ‘Infected + ppIgG’ group than in the infected (no ppIgG) control group (S4A Fig) and in a multivariate analysis by NMDS, there was limited separation between groups, mainly by the ‘Infected + ppIgG’ group being slightly separated from the control groups on the first axis (S4B Fig).

## Discussion

The purified pig IgG product (ppIgG) can be assumed to represent a population of natural antibodies having broad specificities against a vast number of pathogens and epitopes, comparable with Intravenous immunoglobulin (IVIg) preparations widely used for human applications [1]. We hypothesise that such purified immunoglobulin preparations may contain reactivity against a wide selection of pathogens infecting through the enteric route as the ppIgG originates from slaughter swine presumably harbouring a multitude of different anti-pathogenic immunoglobulins reflecting the exposure to many different infectious agents throughout the pig’s life, including diarrhoeagenic *E*. *coli*. Hence, providing these immunoglobulins orally is presumed to inhibit adhesion of bacterial pathogens to the intestinal (epithelial) cell wall by immunoglobulins binding to these bacteria. The ppIgG investigated here contained approximately 85% pure IgG. Our *in vitro* results suggest that substantial amounts of ppIgG need to be supplemented in the diet as at least 6 milligrams of ppIgG were needed to inhibit bacterial adhesion to intestinal epithelial cells. This probably reflects the fact that only a minor part of the natural plasma immunoglobulin pool contains binding activities directed against the specific bacterial species (*E*. *coli* and *S*. *enterica*) investigated here. It was demonstrated that ppIgG does have a preference for binding relevant bacteria such as *E*. *coli* and *Salmonella enterica* having negligible binding to *Y*. *ruckeri*, a fish pathogen with no relevance for pigs. The ppIgG investigated here bound three different *E*. *coli* isolates and one *S*. *enterica* isolate equally well. Also, the anti-bacterial IgG of ppIgG was found to bind strongly to bacterial epitopes as the binding strength (avidity) of ppIgG towards *E*. *coli* was equal to or greater than seen in other studies using urea to interfere with the antibody-antigen binding [24–28]. Thus natural immunoglobulins purified directly from pig plasma (ppIgG) bind strongly to potentially diarrhoeagenic bacteria and inhibit bacterial adhesion to intestinal cells suggesting the use of ppIgG as an antibiotics alternative for prevention and treatment of enteric infections.

The present study establishes the potential of ppIgG purified directly from slaughterhouse blood waste as an antibiotics alternative when used as a feed additive. This supplements earlier studies on the effects of orally provided spray-dried plasma on growth and intestinal infections in both swine and chicken and further underlines the importance of immunoglobulins for the effect of SDPP [15, 29–32]. Furthermore the usage of isolated immunoglobulins from plasma in preventing/treating enteric infections is supported by Pierce *et al* who found that the plasma proteins with high molecular weight (primarily IgG) reduced ETEC infections in weaner piglets [16].

We observed no evidence of any adverse effects in weaner piglets feed with a ppIgG supplement for 14 days in two previous experiments (manuscript in preparation). The 14 day experimental period may be too short to allow potential adverse side effects to manifest; thus, future studies will include observation of any adverse reaction in the pigs until slaughter. Also, in contrast to spray-dried porcine plasma (SDPP) [33, 34] the ppIgG product studied here is not heat treated and, although probably not a problem in IgG purified by EBA chromatography the absence of infectious viral agents will have to be demonstrated (work in progress).

The purified plasma IgG was tested in weaning piglets orally inoculated with an ETEC F4+ isolate. It was found that the faecal clearance of F4+ *E*. *coli* was significantly faster in ppIgG-fed piglets than in the infected control group not being fed ppIgG. Furthermore, deep sequencing of part of the 16S rRNA genes obtained from the total ileal microbiota revealed that the proportion of family *Enterobacteriaceae* was significantly lower in the group of weaners receiving the ppIgG product than in the infected control group not receiving ppIgG and the non-infected control group. Importantly, the *Enterobacteriaceae* family comprises *E*. *coli*. Conversely, the ileal colonization with both *Enterococcoceae* and *Streptococcaceae* was significantly higher in the pigs receiving ppIgG compared to the two control groups. The biological significance of this is uncertain. Enteroccoccus has been shown to be associated with piglet neo-natal diarrhoea, however this appears to require co-colonization with *E*. *coli* [35], which is not the case in the present study. The Shannon-index, a measure of diversity, of the ppIgG-treated animals was higher, suggesting a more diverse microbial community which is generally linked to a healthier and more disease resilient microbiota [36]. Overall, the decreased proportion of *Enterobactericeae* as well as the increased diversity, suggest a direct positive effect on the ileal microbiome by ppIgG and in the context of antibiotic alternatives for ETEC treatment, the ppIgG product characterised in this study shows potential for reducing colonization by ETEC.

Conclusively, our data support that natural IgG directly purified from pig plasma and given as a feed supplement can be used in modern swine production both for reducing the occurrence of PWD and for decreasing antibiotic usage. It is envisaged that the ppIgG product may be used in a targeted way e.g. by providing it with the feed for five days pre-weaning and for ten days post weaning. An alternative strategy could be to provide it to the sow before farrowing and pre-weaning to create a beneficial microbiological environment. Together with the low manufacturing price, achieved by the purification of immunoglobulins by a high-volume and efficient chromatographic process directly from inexpensive and renewable sources this makes the ppIgG product relevant as an alternative to antibiotics for the control of PWD and potentially also for other infectious disease in the modern pig production even if these diseases may be multifactorial or have unknown infectious causes.

## Supporting Information

S1 Fig Wells were coated with deoxycholate extract of *E*. *coli O138* in 2-fold serial dilutions and incubated with 6 mg/ml ppIgG. After incubation, PBS-T with 2M, 4M or 6M urea or without urea was added to designated wells and were allowed to incubate for 10, 20 or 30 minutes as indicated before the urea-solution was washed away. Data is presented as median ± ranges. Two-way ANOVA (subjected to Bonferroni post-test) was used to observe statistical significance between treated and untreated samples, designated as ^a^ (p<0.05); ^b^ (p<0.01); ^c^ (p<0.001).
(TIF)

S2 Fig Wells were coated with formalin-fixed *E*. *coli O138*, and incubated with porcine plasma or ppIgG (purified from the same plasma) added in serial 2-fold dilutions, and the bound IgG was estimated by using a HRP-conjugated goat anti-pig IgG antibody Data from one typical experiment are presented. (TIF)

S3 Fig Aliquots of ppIgG were placed either at room-temperature (RT), refrigerated (4°C) or in freezer (-20°C). At day 35, 65, 90 and 120 after storage one aliquot was taken from each storage place and analysed for its ability to displace the signal in a competitive ELISA (see Materials and Methods) in which the antigens were either from *E*. *coli O138* or *S*. *diarizonae*. Data is presented as median± ranges. Statistical significance in comparison with initial antibody activity was based on two-way ANOVA (subjected to Bonferroni post-test); **p<0.01; *** p< 0.001.
(TIF)

S4 Fig **(A).** Ileal Shannon-index. Bacteria were enumerated by NGS sequencing of the 16S rDNA gene in ileal samples obtained at necropsy by the end of the experiment. Overall ANOVA p-value < 0.001, different letters denote significantly different values. **(B)** Unconstrained multidimensional scaling of the ileal microbiota on a family level, calculated for k = 2 using Bray-curtis distances. Bacteria were enumerated by NGS sequencing of the 16S rDNA gene in ileal samples obtained at necropsy by the end of the experiment.
(TIF)

S1 Table Effect of storage on immunoglobulin bacterial binding. (DOCX)

## References

[1] Kaveri SV. Intravenous immunoglobulin: exploiting the potential of natural antibodies. Autoimmunity reviews. 2012;11(11):792–4. 10.1016/j.autrev.2012.02.006 . 22349620

[2] Hurley WL, Theil PK. Perspectives on immunoglobulins in colostrum and milk. Nutrients. 2011;3(4):442–74. 10.3390/nu3040442 22254105 PMC3257684

[3] Madi A, Bransburg-Zabary S, Kenett DY, Ben-Jacob E, Cohen IR. The natural autoantibody repertoire in newborns and adults: a current overview. Advances in experimental medicine and biology. 2012;750:198–212. 10.1007/978-1-4614-3461-0_15 . 22903676

[4] Levast B, Berri M, Wilson HL, Meurens F, Salmon H. Development of gut immunoglobulin A production in piglet in response to innate and environmental factors. Developmental & Comparative Immunology. 2014;44(1):235–44. 10.1016/j.dci.2013.12.012 . 24384471

[5] Brandtzaeg P. The mucosal immune system and its integration with the mammary glands. The Journal of pediatrics. 2010;156(2 Suppl):S8–15. 10.1016/j.jpeds.2009.11.014 . 20105666

[6] Fairbrother JM, Nadeau E, Gyles CL. Escherichia coli in postweaning diarrhea in pigs: an update on bacterial types, pathogenesis, and prevention strategies. Animal health research reviews / Conference of Research Workers in Animal Diseases. 2005;6(1):17–39. . 16164007 10.1079/ahr2005105

[7] DANMAP2014. Use of antimicrobial agents and occurrence of antimicrobial resistance in bacteria from food animals, food and humans in Denmark Søborg: Technical University of Denmark, Statens Serum Institute; 2014. Available from: http://www.danmap.org/~/media/Projekt%20sites/Danmap/DANMAP%20reports/DANMAP%202014/Danmap_2014.ashx.

[8] WHO. The Evolving Threat of Antimicrobial Resistance—Options for Action: World Health Organisation; 2012 Available from: http://apps.who.int/iris/bitstream/10665/44812/1/9789241503181_eng.pdf.

[9] CDC. Antibiotic Resistance Threats in the United States: Center of Disease Control; 2013 Available from: http://www.cdc.gov/drugresistance/pdf/ar-threats-2013-508.pdf.

[10] ECDC/EMEA. The bacterial challenge: time to react: European Center of Disease Prevention and Control, European Medicines Agency; 2009 54]. Available from: http://www.ema.europa.eu/docs/en_GB/document_library/Report/2009/11/WC500008770.pdf.

[11] Viswanathan VK, Hodges K, Hecht G. Enteric infection meets intestinal function: how bacterial pathogens cause diarrhoea. Nat Rev Microbiol. 2009;7(2):110–9. 10.1038/nrmicro2053 WOS:000263090900010. 19116615 PMC3326399

[12] Rhee JH, Lee SE, Kim SY. Mucosal vaccine adjuvants update. Clinical and Experimental Vaccine Research. 2012;1(1):50–63. 10.7774/cevr.2012.1.1.50 23596577 PMC3623511

[13] Melkebeek V, Goddeeris BM, Cox E. ETEC vaccination in pigs. Veterinary immunology and immunopathology. 2013;152(1–2):37–42. 10.1016/j.vetimm.2012.09.024 . 23068270

[14] EMEA. Coliprotec F4: European Medicines Agency; 2015. Available from: http://www.ema.europa.eu/docs/en_GB/document_library/EPAR_-_Summary_for_the_public/veterinary/003797/WC500184534.pdf.

[15] Torrallardona D. Spray Dried Animal Plasma as an Alternative to Antibiotics in Weanling Pigs—A Review. Asian Austral J Anim. 2010;23(1):131–48. WOS:000273360300019.

[16] Pierce JL, Cromwell GL, Lindemann MD, Russell LE, Weaver EM. Effects of spray-dried animal plasma and immunoglobulins on performance of early weaned pigs. Journal of animal science. 2005;83(12):2876–85. . 16282627 10.2527/2005.83122876x

[17] Niewold TA, van Dijk AJ, Geenen PL, Roodink H, Margry R, van der Meulen J. Dietary specific antibodies in spray-dried immune plasma prevent enterotoxigenic Escherichia coli F4 (ETEC) post weaning diarrhoea in piglets. Veterinary microbiology. 2007;124(3–4):362–9. 10.1016/j.vetmic.2007.04.034 . 17524575

[18] Bhandari SK, Xu B, Nyachoti CM, Giesting DW, Krause DO. Evaluation of alternatives to antibiotics using an Escherichia coli K88(+) model of piglet diarrhea: Effects on gut microbial ecology. Journal of animal science. 2008;86(4):836–47. 10.2527/jas.2006-822 WOS:000254083500006. 18192551

[19] Lihme A, Hansen MB, Andersen IV, Burnouf T. A novel core fractionation process of human plasma by expanded bed adsorption chromatography. Analytical Biochemistry. 2010;399(1):102–9. 10.1016/j.ab.2009.12.002 . 19995544

[20] Boesen HT, Jensen TK, Moller K, Nielsen LH, Jungersen G. Evaluation of a novel enzyme-linked immunosorbent assay for serological diagnosis of porcine proliferative enteropathy. Veterinary microbiology. 2005;109(1–2):105–12. 10.1016/j.vetmic.2005.05.004 . 15975740

[21] Stahl M, Kokotovic B, Hjulsager CK, Breum SO, Angen O. The use of quantitative PCR for identification and quantification of Brachyspira pilosicoli, Lawsonia intracellularis and Escherichia coli fimbrial types F4 and F18 in pig feces. Veterinary microbiology. 2011;151(3–4):307–14. 10.1016/j.vetmic.2011.03.013 WOS:000293428400012. 21530108

[22] Strube ML, Ravn HC, Ingerslev HC, Meyer AS, Boye M. In situ prebiotics for weaning piglets: in vitro production and fermentation of potato galacto-rhamnogalacturonan. Applied and Environmental Microbiology. 2015;81(5):1668–78. 10.1128/AEM.03582-14 25527557 PMC4325168

[23] Cole JR, Wang Q, Fish JA, Chai B, McGarrell DM, Sun Y, et al Ribosomal Database Project: data and tools for high throughput rRNA analysis. Nucleic Acids Res. 2014;42(Database issue):D633–42. 10.1093/nar/gkt1244 24288368 PMC3965039

[24] Chan KH, Sonnenberg K, Niedrig M, Lam SY, Pang CM, Chan KM, et al Use of antibody avidity assays for diagnosis of severe acute respiratory syndrome coronavirus infection. Clin Vaccine Immunol. 2007;14(11):1433–6. 10.1128/Cvi.00056-07 WOS:000251125800007. 17881505 PMC2168165

[25] de Souza VAUF, Fernandes S, Araujo ES, Tateno AF, Oliveira OMNPF, Oliveira RD, et al Use of an immunoglobulin G avidity test to discriminate between primary and secondary dengue virus infections. J Clin Microbiol. 2004;42(4):1782–4. 10.1128/Jcm.42.4.1782-1784.2004 WOS:000220963000072. 15071049 PMC387572

[26] Levett PN, Sonnenberg K, Sidaway F, Shead S, Niedrig M, Steinhagen K, et al Use of immunoglobulin G avidity assays for differentiation of primary from previous infections with West Nile virus. J Clin Microbiol. 2005;43(12):5873–5. 10.1128/Jcm.43.12.5873-5875.2005 WOS:000234055400006. 16333069 PMC1317205

[27] Hoorfar J, Feld NC, Schirmer AL, Bitsch V, Lind P. Serodiagnosis of Salmonella dublin infection in Danish dairy herds using O-antigen based enzyme-linked immunosorbent assay. Canadian journal of veterinary research = Revue canadienne de recherche veterinaire. 1994;58(4):268–74. 7889458 PMC1263711

[28] Jenum PA, Stray-Pedersen B, Gundersen AG. Improved diagnosis of primary Toxoplasma gondii infection in early pregnancy by determination of antitoxoplasma immunoglobulin G avidity. J Clin Microbiol. 1997;35(8):1972–7. 9230365 10.1128/jcm.35.8.1972-1977.1997 PMC229886

[29] Nollet H, Deprez P, Van Driessche E, Muylle E. Protection of just weaned pigs against infection with F18(+) Escherichia coli by non-immune plasma powder. Veterinary microbiology. 1999;65(1):37–45. WOS:000078560100004. 10068126 10.1016/s0378-1135(98)00282-x

[30] Jamroz D, Wiliczkiewicz A, Orda J, Kuryszko J, Stefaniak T. Use of spray-dried porcine blood by-products in diets for young chickens. Journal of animal physiology and animal nutrition. 2012;96(2):319–33. 10.1111/j.1439-0396.2011.01149.x . 21561488

[31] Jamroz D, Wiliczkiewicz A, Orda J, Skorupinska J, Slupczynska M, Kuryszko J. Chemical composition and biological value of spray dried porcine blood by-products and bone protein hydrolysate for young chickens. British poultry science. 2011;52(5):589–605. 10.1080/00071668.2011.610298 . 22029787

[32] Campbell JM, Russell LE, Crenshaw JD, Behnke KC, Clark PM. Growth response of broilers to spray-dried plasma in pelleted or expanded feed processed at high temperature. Journal of animal science. 2006;84(9):2501–8. 10.2527/jas.2005-722 . 16908655

[33] Opriessnig T, Xiao CT, Gerber PF, Zhang J, Halbur PG. Porcine epidemic diarrhea virus RNA present in commercial spray-dried porcine plasma is not infectious to naive pigs. Plos One. 2014;9(8):e104766 10.1371/journal.pone.0104766 25116479 PMC4130536

[34] Shen HG, Schalk S, Halbur PG, Campbell JM, Russell LE, Opriessnig T. Commercially produced spray-dried porcine plasma contains increased concentrations of porcine circovirus type 2 DNA but does not transmit porcine circovirus type 2 when fed to naive pigs. Journal of animal science. 2011;89(6):1930–8. 10.2527/jas.2010-3502 . 21278103

[35] Hermann-Bank ML, Skovgaard K, Stockmarr A, Strube ML, Larsen N, Kongsted H, et al Characterization of the bacterial gut microbiota of piglets suffering from new neonatal porcine diarrhoea. BMC veterinary research. 2015;11:139 10.1186/s12917-015-0419-4 26099928 PMC4476181

[36] Lozupone CA, Stombaugh JI, Gordon JI, Jansson JK, Knight R. Diversity, stability and resilience of the human gut microbiota. Nature. 2012;489(7415):220–30. 10.1038/nature11550 22972295 PMC3577372

