# Supplementary figures and images for: Natural Pig Plasma Immunoglobulins Have Anti-Bacterial Effects: Potential for Use as Feed Supplement for Treatment of Intestinal Infections in Pigs
Source: PLoS One. 2016 Jan 29;11(1):e0147373. doi: 10.1371/journal.pone.0147373 (PMC4744083; doi:10.1371/journal.pone.0147373)

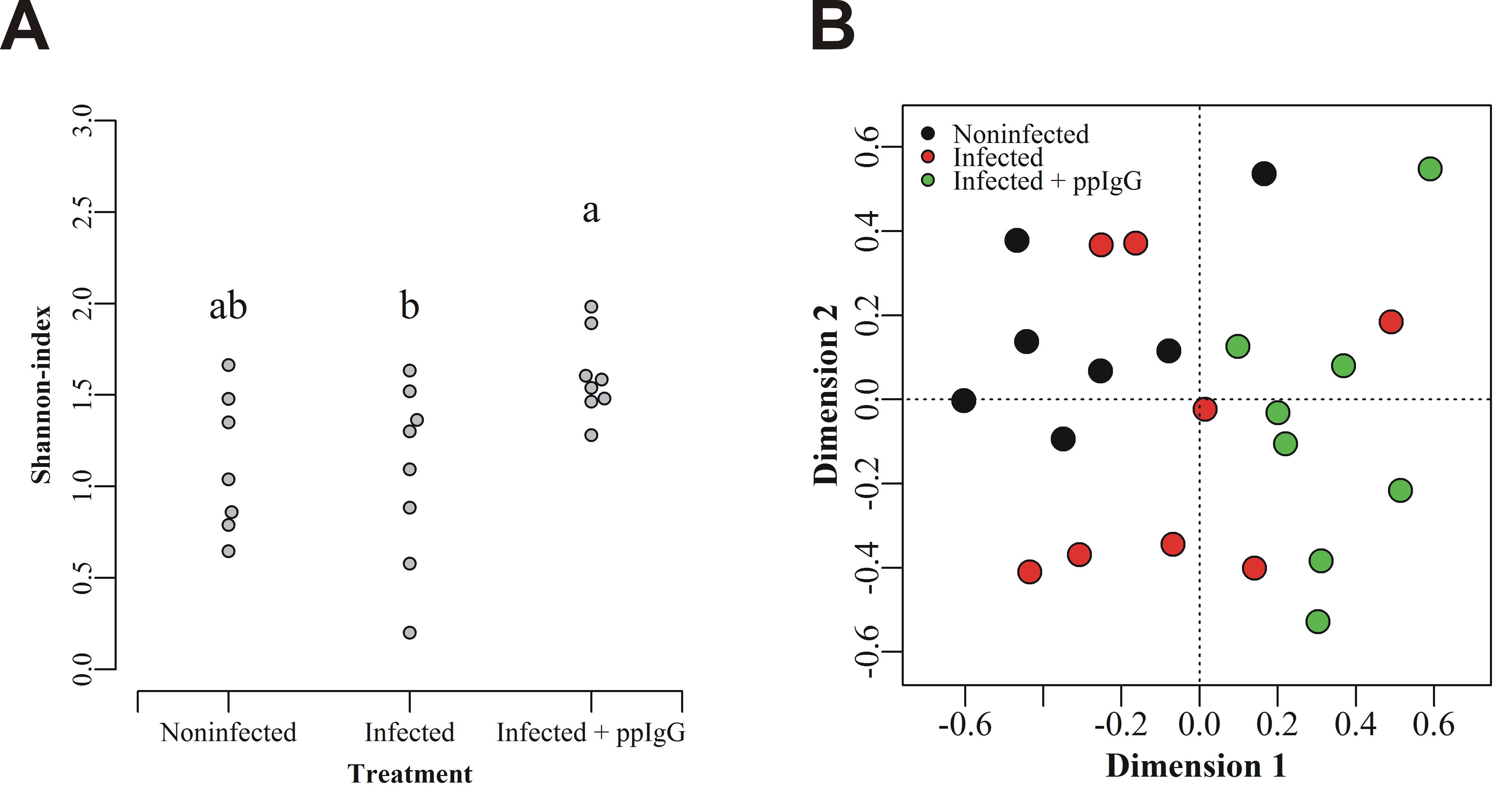

Supplement: S4 Fig — (A). Ileal Shannon-index. Bacteria were enumerated by NGS sequencing of the 16S rDNA gene in ileal samples obtained at necropsy by the end of the experiment. Overall ANOVA p-value < 0.001, different letters denote significantly different values. (B) Unconstrained multidimensional scaling of the ileal microbiota on a family level, calculated for k = 2 using Bray-curtis distances. Bacteria were enumerated by NGS sequencing of the 16S rDNA gene in ileal samples obtained at necropsy by the end of the experiment. (TIF) [file pone.0147373.s004.tif]
