# Supplementary material for: Natural Pig Plasma Immunoglobulins Have Anti-Bacterial Effects: Potential for Use as Feed Supplement for Treatment of Intestinal Infections in Pigs
Source: PLoS One. 2016 Jan 29;11(1):e0147373. doi: 10.1371/journal.pone.0147373 (PMC4744083; doi:10.1371/journal.pone.0147373)
Supplement: S1 Table — (DOCX) [file pone.0147373.s005.docx]

|  | **% of original reactivity** | | | | | |
| --- | --- | --- | --- | --- | --- | --- |
|  | ***E. coli*** | | | ***S. enterica*** | | |
| **Days/Storage** | Room temperature | 4°C | -20°C | Room temperature | 4°C | -20°C |
| **35** | 68.4 | 78.6 | 91.2 | 91.6 | 88.0 | 89.7 |
| **65** | 53.2 | 66.1 | 82.9 | 89.6 | 83.1 | 95.3 |
| **90** | Below assay limit | 41.2 | 80.1 | 51.9 | 73.2 | 91.2 |
| **120** | Below assay limit | 42.2 | 81.8 | 32.5 | 53.6 | 91.6 |
